# Cost-effectiveness analysis of apixaban versus vitamin K antagonists for antithrombotic therapy in patients with atrial fibrillation after acute coronary syndrome or percutaneous coronary intervention in Spain

**DOI:** 10.1371/journal.pone.0259251

**Published:** 2021-11-12

**Authors:** Simone Rivolo, Manuela Di Fusco, Carlos Polanco, Amiee Kang, Devender Dhanda, Mirko Savone, Aristeidis Skandamis, Thitima Kongnakorn, Javier Soto

**Affiliations:** 1 Modeling and Simulation, Evidera PPD, Milan, Italy; 2 Pfizer Inc, New York, New York, United States of America; 3 Bristol Myers Squibb, Madrid, Spain; 4 Bristol Myers Squibb, Lawrenceville, New Jersey, United States of America; 5 Modeling and Simulation, Evidera, London, United Kingdom; 6 Pfizer, Madrid, Spain; Leiden University Medical Center, NETHERLANDS

## Abstract

**Background/Objective:**

AUGUSTUS trial demonstrated that, for patients with atrial fibrillation (AF) having acute coronary syndrome (ACS) or undergoing percutaneous coronary intervention (PCI), an antithrombotic regimen with apixaban and P2Y12 resulted in less bleeding, fewer hospitalizations, and similar ischemic events than regimens including a vitamin K antagonist (VKA), aspirin, or both. This study objective was to evaluate long-term health and economic outcomes and the cost-effectiveness of apixaban over VKA, as a treatment option for patients with AF having ACS/PCI.

**Methods:**

A lifetime Markov cohort model was developed comparing apixaban versus VKA across multiple treatment strategies (triple [with P2Y12 + aspirin] or dual [with P2Y12] therapy followed by monotherapy [apixaban or VKA]; triple followed by dual and then monotherapy; dual followed by monotherapy). The model adopted the Spanish healthcare perspective, with a 3-month cycle length and costs and health outcomes discounted at 3%.

**Results:**

Treatment with apixaban resulted in total cost savings of €883 and higher life years (LYs) and quality-adjusted LYs (QALYs) per patient than VKA (net difference, LYs: 0.13; QALYs: 0.11). Bleeding and ischemic events (per 100 patients) were lower with apixaban than VKA (net difference, –13.9 and –1.8, respectively). Incremental net monetary benefit for apixaban was €3,041, using a willingness-to-pay threshold of €20,000 per QALY. In probabilistic sensitivity analysis, apixaban was dominant in the majority of simulations (92.6%), providing additional QALYs at lower costs than VKA.

**Conclusions:**

Apixaban was a dominant treatment strategy than VKA from both the Spanish payer’s and societal perspectives, regardless of treatment strategy considered.

## Introduction

Atrial fibrillation (AF) is the most common form of cardiac dysrhythmia associated with substantial morbidity and mortality with increasing age [1, 2]. In Europe, number of newly diagnosed patients with AF per year ranges from 78,000 to 116, 000 [3]. The current prevalence of AF is estimated to be >11 million, and is projected to be over 17.9 million by 2060 [3, 4]. Acute coronary syndrome (ACS) and acute myocardial infarction (MI) are often associated with AF due to common risk factors [2, 5]. Hence, patients with AF having ACS or undergoing percutaneous coronary intervention (PCI) are more likely to experience complications [6–8] and mortality [9, 10], thus incurring substantial total healthcare costs [11].

Clinical management of AF and ACS, although different, includes antithrombotic therapy to prevent increased risk of stroke and further cardiac events [1, 4, 12]. Oral anticoagulants (OACs), which can either be vitamin K antagonists (VKAs; e.g., warfarin) or novel oral anticoagulants (NOACs; e.g., apixaban, dabigatran, edoxaban, rivaroxaban), are used for AF treatment, whereas dual antiplatelet therapy (DAPT) consisting of aspirin and P2Y12 inhibitors (e.g., clopidogrel) is used for ACS treatment [1, 4, 12, 13]. The optimal antithrombotic regimen for patients with AF having ACS/PCI remains a clinical conundrum due to an increased risk of major and fatal bleedings associated with a combination of OAC and DAPT (i.e., triple therapy) [1, 4, 12, 13]. Moreover, there is limited guidance on the optimal strategy and lack of evidence for all possible combinations of novel antiplatelet and anticoagulant agents.

Several trials in patients with AF having ACS/PCI attempted to address this conundrum. The WOEST trial [14] demonstrated significant reduction in bleeding and no increase in the rate of thrombotic events in patients treated with dual therapy (clopidogrel and VKA) than with triple therapy (clopidogrel, VKA, and aspirin). Three more recent NOAC trials (PIONEER AF-PCI [rivaroxaban], RE-DUAL [dabigatran], and ENTRUST-AF PCI [edoxaban]) demonstrated that compared with VKA triple therapy (i.e., VKA and DAPT), NOAC dual (NOAC and clopidrogrel) or triple therapy (NOAC and DAPT), was either non-inferior or led to a significant reduction in bleeding, without significant differences in ischemic events [15–18]. Although these findings encourage the use of NOACs over VKA, because of their trial design, it was unclear whether reduced bleeding was due to the use of an NOAC or removal of aspirin from the treatment regimen [19].

AUGUSTUS, a prospective, multicenter, open-label, two-by-two randomized controlled trial, evaluated the clinical benefits of dual or triple therapy with apixaban versus VKA and aspirin versus placebo in patients with AF having ACS/PCI. This two-by-two factorial design of the trial also enabled to investigate the impact of including aspirin to the OAC plus P2Y12 treatment regimen, regardless whether apixaban or VKA was the OAC of choice. At 6 months, incidence of bleeding and death or hospitalization were lower with treatment regimens having apixaban, without aspirin, than regimens that had VKA, aspirin, or both, without significant differences in the incidence of ischemic events [20].

No specific guidelines focusing on treatment strategy for patients with AF having ACS/PCI have been published. However, recently, European Society of Cardiology (ESC) guidelines were updated based on the outcome of the clinical trials discussed above [15–18, 20]. The guidelines recommend dual antithrombotic therapy including OAC (NOAC plus P2Y12) for 6–12 months, followed by long-term monotherapy with NOACs. Triple therapy (with aspirin) up to 1 month only is suggested for patients at increased risk of ischemic events [1, 4, 12, 13].

While the AUGUSTUS trial [20] demonstrated the clinical benefits of dual or triple therapy with apixaban over VKA, these benefits will need to be weighed against the economic consequences. In fact, multiple stakeholders (clinicians, payers, patients) would be interested in understanding the economic implications of different treatment options (e.g., apixaban vs VKA) across the multiple available treatment strategies. However, to the best of our knowledge, no cost-effectiveness analyses based on the AUGUSTUS trial have been published. Therefore, the objective of the present study was to investigate the long-term health and economic outcomes and the cost-effectiveness of apixaban compared with VKA, as a treatment option for patients with AF having ACS/PCI, from the Spanish healthcare system perspective. The Spanish perspective was chosen since VKA remains a treatment option commonly used, despite the recent introduction of NOACs [21, 22].

## Methods

### Model overview and structure

A targeted literature review of existing economic evaluations in AF and ACS was performed to conceptualize the cost-effectiveness model and guide decision of model structure and modelling assumptions [23–29]. Similar to the previous economic studies, a Markov cohort approach was used (Further details are provided in the S1 File) with a 3-month cycle length. The modelled cohort represented patients with non-valvular AF (NVAF) starting triple or dual therapy within 14 days after having ACS or undergoing PCI (or both), aligned with the AUGUSTUS trial population [19, 20]. The model enabled comparison of apixaban versus VKA across multiple treatment strategies: i) patients started on triple or dual therapy (triple or dual), then switched to OAC monotherapy after 6 months; ii) patients started on triple therapy (triple), then switched to dual therapy (dual) after 3 months, and then to OAC monotherapy after 6–9 months; iii) patients started on dual therapy, then switched to OAC monotherapy after 6–12 months. Dual therapy included OAC (apixaban, VKA) in combination with P2Y12 (clopidogrel, prasugrel, ticagrelor) while triple therapy also considered aspirin in combination with the dual therapy regimen (S1 Table). A lifetime horizon (up to 100 years) was adopted for the analysis, to capture all relevant differences in future costs and outcomes from the different treatment alternatives being considered, in line with previous economic models [23, 28, 29].

Briefly, at each model cycle (see S1 Fig), patients could experience MI, ischemic stroke (IS), intracranial hemorrhage (ICH), or other major bleeds (OMB). These events were modelled as acute health states (see S2 Fig) with long-term impact in terms of healthcare costs, patients’ health-related quality of life, and risk of subsequent events, in agreement with previously reported studies [23, 24, 28]. The model captured up to two concomitant events (i.e., joint health states–see S3 Fig). Patients experiencing a second clinical event incurred acute cost and acute disutility specific to the second event along with post-acute cost and disutility of the two concomitant events. Clinically relevant non-major bleeding (CRNMB), urgent revascularization (REV), and systemic embolism (SE) were considered short-term events with the proportion of patients experiencing the event accruing a one-off (one-cycle) cost and short-term disutility. Death could have occurred from each health state at each model cycle, either related to the clinical event or due to the excess mortality associated with the clinical events history. Further details on model structure are provided in the S1 File.

### Treatment switching and discontinuation

The model assumed that treatment switching would happen at fixed time points (e.g., switching from dual to monotherapy at 6 months) and no backward transitions were allowed once a patient stepped-down the therapy.

The model also captured treatment discontinuation related or unrelated to clinical events. Patients experiencing a clinical event had a probability of discontinuing to no-treatment [23] or interrupt treatment for a pre-specified period. The treatment interruption was assumed to impact only the cost and not the risk of subsequent events [25–27]. Patients in the post-acute health states had a probability of discontinuing the treatment, at each model cycle, for reasons unrelated to clinical event, as observed in the AUGUSTUS trial [20]. Upon treatment discontinuation, patients were assumed to remain off-treatment for the rest of the time horizon.

### Model inputs

Patient demographics, clinical (Table 1), and cost inputs (Table 2) were obtained primarily from clinical trials and published literature.

**Table 1:** Model demographic and clinical inputs and data sources.

		OMB	CRMNB	MI	IS	ICH	REV	SE
**Demographic inputs**								
Age (years), mean (standard error): 69.9 (0.13) [20]–varied using normal distribution in PSA								
Gender, n (%): male (4,614 [71.0]) [20]–varied using beta distribution in PSA								
**Clinical inputs**								
Triple or dual, triple, and dual event rates per 100 patient years (standard error^a^) [20]–varied using gamma distribution in PSA								
Triple or dual^b^	Apixaban	6.25 (0.02)	18.25 (0.03)	6.65 (0.02)	1.18 (0.01)	0.50 (0.005)	5.63 (0.02)	0.09 (0.00)^c^
	VKA	9.23 (0.02)	26.07 (0.03)	7.44 (0.02)	2.38 (0.01)	1.30 (0.01)	5.94 (0.02)	0.10 (0.00)^c^
Triple^d^	Apixaban	8.98 (0.04)	24.90 (0.07)	6.29 (0.03)	1.46 (0.02)	0.72 (0.01)	4.80 (0.03)	0.09 (0.00)^c^
	VKA	11.66 (0.05)	36.40 (0.09)	6.31 (0.03)	2.21 (0.02)	1.64 (0.02)	5.38 (0.03)	0.10 (0.00)^c^
Dual^d^	Apixaban	4.17 (0.03)	12.30 (0.05)	7.01 (0.04)	0.91 (0.01)	0.33 (0.01)	6.46 (0.03)	0.09 (0.00)^c^
	VKA	8.06 (0.04)	19.00 (0.06)	8.57 (0.04)	2.56 (0.02)	1.14 (0.01)	6.50 (0.03)	0.10 (0.00)^c^
Monotherapy event rates per 100 patient years (standard error^e^)–varied using gamma distribution in PSA								
Apixaban		2.12 (0.003) [30]	2.08 (0.001) [31, 32]	0.95 (0.002) [30]	0.97 (0.001) [32]	0.27 (0.001) [30]	1.69 (0.003) [30]	0.09 (0.001) [32]
Warfarin		2.32 (0.004) [30]	2.99 (0.001) [31, 32]	1.00 (0.002) [30]	1.05 (0.001) [32]	0.73 (0.002) [30]	1.89 (0.003) [30]	0.10 (0.001) [32]
Event rates: apixaban vs no treatment, HR (95% CI)^f^ –varied using LogNormal distribution in PSA								
		1.24 (0.70–2.26) [33]	1.24 (0.70–2.26)^g^	0.44 (0.20–1.03) [33]	0.26 (0.18–0.37) [33]	1.90 (0.64–6.49) [33]	0.44 (0.20–1.03)^h^	0.26 (0.18–0.37)^i^
Increased risk of event due to aging, per decade of life, HR (95% CI) [31]–varied using LogNormal distribution in PSA								
		1.97 (1.79–2.16)	–	1.30 (0.74–2.01)	1.46 (0.80–2.16)	1.97 (1.79–2.16)	–	–
Increased risk of subsequent events, HR (95% CI) [34]–varied using LogNormal distribution in PSA								
Experiencing future ICH		3.54 (3.02–4.17)	–	1.00	1.64 (1.39–1.94)	10.20 (8.59–12.20)	–	–
Experiencing future OMB		3.32 (3.06–3.60)	–	1.00	1.39 (1.27–1.52)	2.95 (2.57–3.39)	–	–
Experiencing future MI^j^		1.00	–	1.00	1.00	1.00	–	–
Experiencing future IS		1.32 (1.21–1.44)	–	1.00	4.00 (3.78–4.22)	1.78 (1.56–2.03)	–	–
Probability (%) of discontinuation per event (standard error) [31]–varied using beta distribution in PSA								
		25.0 (2.50)	–	0.0 (NA)	0.0 (NA)	55.8 (6.89)	–	–
Probability (%) of discontinuation unrelated to clinical events (per cycle) (standard error) [31]–varied using beta distribution in PSA								
Apixaban		4.54 (0.45)						
VKA		4.80 (0.48)						
Post-acute event mortality risk vs general population; Country, HRs (95% CI)^k^ –varied using LogNormal distribution in PSA								
		–	–	UK, 1.45 (1.38–1.53)^l^^,^^m^ [35]	US, 2.60 (2.30–3.00)^n^ [36]	Finland, 2.20 (NR)^o^ [37]	–	–
Mortality risk for each long-term event–varied using beta distribution in PSA								
		Age (years)		Treatment interval (days)		CFR (%)		–
ICH [38]		65–75		30		18.10		–
		75–85		30		26.80		–
		> 85		30		30.90		–
OMB [31]		70		90		2.00		–
MI [39]		60–70		30		7.10		–
		70–80		30		10.90		–
		> 80		30		31.60		–
IS [40]		70		90		10.90		–

Abbreviations: ACS = acute coronary syndrome; AF = atrial fibrillation; CI = confidence interval; CRNMB = clinically relevant non-major bleeding; CSR = clinical study report; HR = hazard ratio; ICH = intracranial hemorrhage; IS = ischemic stroke; ISTH = International Society on Thrombosis and Hemostasis; MI = myocardial infarction; NA = not applicable; NR = not reported; OMB = other major bleeds; PCI = percutaneous coronary intervention; PSA = probabilistic sensitivity analysis; REV = urgent revascularization; SE = systemic embolism; UK = United Kingdom; US = United States; VKA = vitamin K antagonist.

^a^Standard errors were based on event count and person years, as they were not available from the AUGUSTUS trial [20] or CSR.

^b^Rates of OMB were calculated by subtracting the event rate of ICH bleeds from the event rate of the ISTH major bleeds.

^c^Standard error for SE was 0.001.

^d^ICH and OMB rates for dual and the triple therapies have been derived from the CSR, using ISTH major bleeds specific to “triple or dual” therapy and assuming the same distribution between OMB and ICH as observed in the “triple or dual” analysis.

^e^Standard errors were based on event count and person years.

^f^HR per decade of life was applied to each model cycle by multiplying the event risk by the cycle-adjusted HR.

^g^Assumed same as OMB.

^h^Assumed same as MI.

^i^Assumed same as IS.

^j^Based on results from a published study (doi: 10.1161/JAHA.117.007267), a conservative HR of 1.00 was applied to future events of MI, following an IS, ICH, OMB, or MI.

^k^Since the modelled cohort consisted of patients with prior ACS/PCI, an HR (95% CI) of 1.16 (1.10–1.22) among patients with history of angina was assumed for the event-free health state.

^l^Participants with a history of acute MI.

^m^Gitsels et al. 2017 [35] reported HRs by age as follows: 1.5 at 70 years and 1.45 at 75+ years. The lowest HR was used for simplicity and conservatism.

^n^Patients with AF were followed up over 6 years and IS events occurring during this time were identified.

^o^Adult patients with first ever ICH.

**Table 2:** Model cost inputs and data sources.

	Apixaban [20]		VKA (acenocoumarol^j^) [31, 41]		Aspirin [20, 41]		Clopidogrel [41]		Prasugrel		Ticagrelor [41]		–	
Drug acquisition costs^a^ [41]–varied using gamma distribution in PSA														
Price per mg (€)	0.19		0.03		0.00		0.01		0.07		0.02		–	
Daily dosage (mg)	10		5^b^		81		75		10		120		–	
Cost per 3 months (€)	173.49		13.70		3.58		54.82		63.92		194.81		–	
P2Y12 distribution (%)^c^ [20]–varied using Dirichlet distribution in PSA														
Apixaban	–		–		–		93.4		1.2		5.4		–	
VKA	–		–		–		91.8		1.1		7.1		–	
Event/maintenance costs–varied using gamma distribution in PSA														
	OMB		CRMNB		MI		IS		ICH		REV		SE	
Acute event costs (€), DRG details [25–27, 41]–varied using gamma distribution in PSA														
	3,341.59^d^		2,412.85^e^		4,153.47, DRG code 190		5,094.95, DRG code 045		5,987.77, DRG code 044		15,599.15, DRG code 166		3,875.89, DRG code 046	
Monthly maintenance costs (€) [25–27, 41]–varied using gamma distribution in PSA														
	0.00^f^		–		168.12		1,494.37		1,494.37		–		–	
Societal event costs; acute phase costs, maintenance phase costs (monthly)^g^—varied using gamma distribution in PSA														
	0, 0		0, NA		0, 0		516.37, 1,610.38		516.37, 1,610.38		0, NA		0, NA	
Utilities–varied using beta distribution in PSA														
For short- and long-term events														
	Event	Control	Event	Control	Event	Control	Event	Control	Event	Control	Event	Control	Event	Control
Mean utility (standard error)	0.808 (0.014) [42]	0.837 (0.001) [42]	0.826 (0.007) [42]	0.836 (0.002) [42]	0.690 (0.011) [43]	0.805 (0.081)^i^ [43]	0.640 (0.016) [44]	0.830 (0.012) [44]	0.560 (0.077) [44]	0.830 (0.012) [44]	0.780 (0.016) [45]	0.800 (0.012) [45]	0.730 (0.014) [44]	0.830 (0.012) [44]
Event: control ratio^h^	0.965 [42]		0.988 [42]		0.857 [43]		0.771 [44]		0.675 [44]		0.975 [45]		0.879 [44]	
Post-acute period														
Mean utility (standard error)	0.829 (0.083) [42]	0.837 (0.001) [42]	–	–	0.702 (0.006) [43]	0.799 (0.080)^i^ [43]	0.685 (0.008) [44]	0.830 (0.012) [44]	0.705 (0.044) [44]	0.830 (0.012) [44]	–	–	–	–
Event: control ratio	0.990 [42]		–		0.878 [43]		0.825 [44]		0.849 [44]		–		–	

Abbreviations: CRNMB = clinically relevant non-major bleeding; CVD = cardiovascular disease; DRG = diagnosis-related group; EMA = European Medicines Agency; EQ-5D = European quality of life 5-dimensions; ICH = intracranial hemorrhage; IS = ischemic stroke; ISTH = International Society on Thrombosis and Hemostasis; MI = myocardial infarction; NA = not applicable; OMB = other major bleeds; PSA = probabilistic sensitivity analysis; REV = urgent revascularization; SE = systemic embolism; UK = United Kingdom; VKA = vitamin K antagonist.

^a^Treatment dosages were derived from the AUGUSTUS trial [20] and EMA labels (Clopidogrel: https://www.ema.europa.eu/en/documents/product-information/plavix-epar-product-information_en.pdf; Prasugrel: https://www.ema.europa.eu/en/documents/product-information/efient-epar-product-information_en.pdf; Ticagrelor: https://www.ema.europa.eu/en/documents/product-information/brilique-epar-product-information_en.pdf).

^b^An average daily dose of 5 mg was assumed (same as in Dorian et al. 2014 [31]).

^c^Distribution of P2Y12 (used in triple or dual treatment regimen) was derived from the AUGUSTUS trial [20]. The same distribution was assumed for the triple and the dual treatment regimen as well.

^d^Weighted average of gastrointestinal and other ISTH major bleeding costs were calculated by number of episodes from DRG codes: 082, 351, 254, 346, 351, 207, and 253.

^e^Weighted average of severity level 1 CRNMB costs were calculated by number of episodes from DRG codes: 468, 115, 253, 144, 661, 663, 143, and 532.

^f^Assumed that no maintenance costs were incurred.

^g^Sourced from Baron Esquivias et al. 2015 [25], that sourced the costs from Beguiristain et al. 2005 (https://www.neurologia.com/articulo/2004436/eng). It was unclear in which of the two studies the assumption of equal costs for ICH and IS were made.

^h^Event to control ratio was used to scale the baseline cohort utility for the proportion of patients experiencing the event.

^i^Estimated based on UK no CVD equation (EQ-5D = 0.9454933 + 0.0256466*male– 0.0002213 × age– 0.0000294 × age^2) by Ara and Brazier 2010 [46], since the values were not available from Pockett et al. 2018 [43].

^j^Note that acenocoumarol efficacy was assumed equivalent to the efficacy of warfarin. Acenocoumarol was considered as the only VKA used in the base case analysis given it is the most common VKA utilized in Spain.

#### Clinical inputs

The event rates (per 100 patient years) for triple or dual, triple, dual therapy (Table 1) were obtained from the AUGUSTUS trial or clinical study report (CSR) of the AUGUSTUS trial [20]. The event rates for monotherapy (Table 1) were obtained from a post-hoc analysis of data from the ARISTOTLE trial among patients with prior coronary artery disease (CAD) [30], as patient characteristics were comparable. Event rates for CRNMB, IS, and SE were derived from the ARISTOTLE trial among overall population [31, 32]. Inputs on event rates for apixaban versus no treatment were derived from Tawfik et al. [33] or clinical similarity. Increased risk of events due to aging was not considered for the first 6 months, because they were already captured in the AUGUSTUS trial [20]. Data for beyond 6 months were obtained from Dorian et al. for consistency with previous model in NVAF [31]. Increased risks for subsequent events were obtained from Friberg et al. [34]. The estimates for the risk of a subsequent MI following a clinical event was not available, thus the model adopted a conservative hazard ratio (HR) of 1.00. Acenocoumarol was considered as the only VKA used in the base case analysis given it is the most common VKA utilized in Spain [47, 48]. Therapeutic equivalence of warfarin (used as VKA in the AUGUSTUS [20] and ARISTOTLE trials [32]) and acenocoumarol was assumed, as commonly done across all Spanish cost-effectiveness studies [25–27]. This assumption is supported by recent clinical evidence [49].

Patients were assumed to start on triple or dual therapy [20], and then switch to monotherapy after 6 months, whereas alternate treatment strategies were evaluated in scenario analysis [1, 4, 12]. Inputs for treatment discontinuation related to clinical events were derived from Dorian et al. [31], whereas scenario analysis evaluated different values used by Sterne et al. [23]. Probabilities of treatment discontinuation (per cycle) unrelated to clinical events were derived by calculating the difference between overall discontinuation rates reported in the AUGUSTUS trial [20], event-related discontinuation based on number of events in the AUGUSTUS trial and discontinuation rates from Dorian et al. [31] and observed death-related discontinuations. The risk of mortality (i.e., case fatality rate [CFRs]) associated with each clinical event (stratified by age) were derived from different studies [31, 38–40]. For the post-acute health states, the Spanish background mortality was adjusted by HRs depending on the presence of co-morbidities, mostly derived from non-Spanish studies, as no Spain-specific studies were available [35–37] (Table 1).

#### Cost inputs

Drug acquisition costs were obtained from Ministerio de Sanidad, Consumo y Bienestar Social 2019 [41]. Since apixaban does not require monitoring, the model captured monitoring costs (€7.05 per international normalized ratio analysis) only for VKA [50]. VKA unit cost in the base case was based on acenocoumarol unit cost of €0.03 per mg (Table 2). As scenario analysis, warfarin unit cost of €0.02 per mg was used for VKA unit cost.

Costs (post-acute and maintenance) related to events were derived from previous Spanish economic studies [25–27]. Acute event costs were derived by the relevant all patient refined-diagnosis-related group (DRG) codes [41]. Evidence on societal costs are limited and were available only for a few events [25]. All unit costs used in the model were inflated to 2019 values if the utilized source reported their estimates for previous costing years [50]. Utilities measured using the European quality of life 5-dimensions valued using the Spanish preferences are generally lacking for the clinical events modelled in this analysis. Hence, the United Kingdom (UK) preferences were mostly used. Inputs for acute and long-term (acute and post-acute period) events were derived from various published studies [42–45] (Table 2). The baseline utility for the modelled cohort was derived from the general population equation provided by Ara and Brazier [46], with the baseline cohort utility decreasing over time due to aging. The event-related decrements were then calculated as the ratio of event cohort over control cohort and applied multiplicatively [46].

### Analyses

#### Base case analysis

The base case analysis focused on treatment strategy starting from triple or dual therapy (with 50% patients on triple and the remainder on dual for 6 months), as per the AUGUSTUS trial [20] and then switching to monotherapy after 6 months (S2 File, Table 1). Furthermore, as per the AUGUSTUS trial [20], 61.2% of patients started in the post-acute MI health state, whereas the remainder in the event-free health state. The costs and health outcomes were discounted at 3% annual rate [51]. The analysis considered direct medical costs from the Spanish healthcare payer’s perspective and direct non-medical and indirect costs from the societal perspective. Increase in event risk (e.g., ICH, OMB, MI, IS) due to aging were included after the first 6 months, whereas patients off-treatment experiencing an event were assumed not to incur additional treatment costs.

#### Deterministic sensitivity analysis

In the deterministic sensitivity analysis (DSA), the lower and upper bounds of each parameter were varied, one by one, based on the 95% upper and lower confidence intervals (CIs). The bounds were derived assuming ± 10% of the base case value, if no CI was available or it was not possible to derive them from other available parameters (Table 2).

#### Probabilistic sensitivity analysis

Joint uncertainty of all key parameters was evaluated by probabilistic sensitivity analysis (PSA), simultaneously sampling the model parameters from the parameter-specific distribution, based on the mean parameter value and associated standard error. This process was repeated 1,000 times to obtain a distribution of incremental costs and effects between treatment groups. The inputs were assumed to follow beta distribution for binary outcomes the gamma distribution for event rates and costs, the lognormal distribution for HRs, and the Dirichlet distribution for multinomial outcomes, as reported earlier [52].

#### Scenario analysis

The impact of different potential assumptions (e.g., alternative treatment strategies, shorter time horizon) or input sources were evaluated across multiple scenarios using scenario analysis.

## Results

### Base case analysis

Results of the base case analysis are presented in Table 3. Treatment with apixaban demonstrated lower costs and higher QALYs than VKA from both payer’s and societal perspectives. In terms of clinical events, bleeding and ischemic events (per 100 patients) were lower with apixaban than VKA (net difference: –13.9 and –1.8, respectively) over the simulated time horizon. Lower counts of bleeding events for apixaban were observed for ICH (net difference: –3.3), CRNMB (– 6.9), and OMB (–3.6).

**Table 3:** Base case analysis results.

	Apixaban	VKA	Net difference (apixaban vs VKA)^a^
**Clinical events (events count per 100 patients)**			
Bleeding events incurred	102.7	116.6	−13.9
ICH	4.5	7.7	−3.3
OMB	66.2	69.9	−3.6
CRNMB	32.0	39.0	−6.9
Ischemic events incurred	135.2	137.0	−1.8
MI	30.0	30.4	−0.4
IS	61.3	61.9	−0.5
REV	40.7	41.5	−0.8
SE	3.2	3.2	−0.0^b^
**Total costs (per patient), €**			
Treatment costs	2,763	346	2,417
Monitoring costs	0	419	−419
Direct costs			
Clinical event costs	43,306	46,187	−2,881
Acute costs	10,884	11,514	−630
Post-acute costs	32,422	34,673	−2,251
Total costs–payer’s perspective	46,069	46,953	−883
Indirect costs			
Clinical event costs	22,711	25,450	−2,740
Acute costs	250	269	−19
Post-acute costs	22,460	25,182	−2,722
Total costs–societal perspective	68,780	72,403	−3,623
**Health outcomes**			
Total LYs	9.88	9.75	0.13
Total QALYs	6.64	6.53	0.11
Time-on-treatment (years)	3.78	3.54	0.23
Time-off-treatment (years)	6.10	6.20	−0.10

Abbreviations: CRNMB = clinically relevant non-major bleeding; ICH = intracranial hemorrhage; IS = ischemic stroke; LYs = life years; MI = myocardial infarction; OMB = other major bleeds; QALYs = quality-adjusted life years; REV = urgent revascularization; SE = systemic embolism; VKA = vitamin K antagonist.

^a^Values for some of the events are slightly different due to rounding off.

^b^SE count difference was 0.05 in favor of apixaban (3.18 vs 3.23).

An incremental cost of €2,417 associated with apixaban treatment, compared with VKA, was offset by savings in monitoring costs (€0 vs €419) as well as lower direct costs related to clinical events (both acute and post-acute) (€43,306 vs €46,187). This led to a total cost saving of €883 per patient with apixaban treatment from payer’s perspective. Furthermore, lower indirect costs related to clinical events with apixaban treatment than VKA (€22,711 vs €25,450) also improved total cost savings to €3,623 per patient from the societal perspective.

Results of health outcomes indicated that total LYs and QALYs were greater with apixaban (net difference: 0.13 and 0.11, respectively), with patients on apixaban experiencing longer treatment duration (net difference: 0.23).

Apixaban was estimated to achieve an incremental net health benefit of 0.152 and 0.289 and incremental net monetary benefit (INMB) of €3,041 and €5,781 from payer’s and societal perspectives, vs VKA, respectively, for a willingness-to-pay (WTP) threshold of €20,000 per QALY in Spain [27].

### Deterministic sensitivity analysis

**Fig 1:**
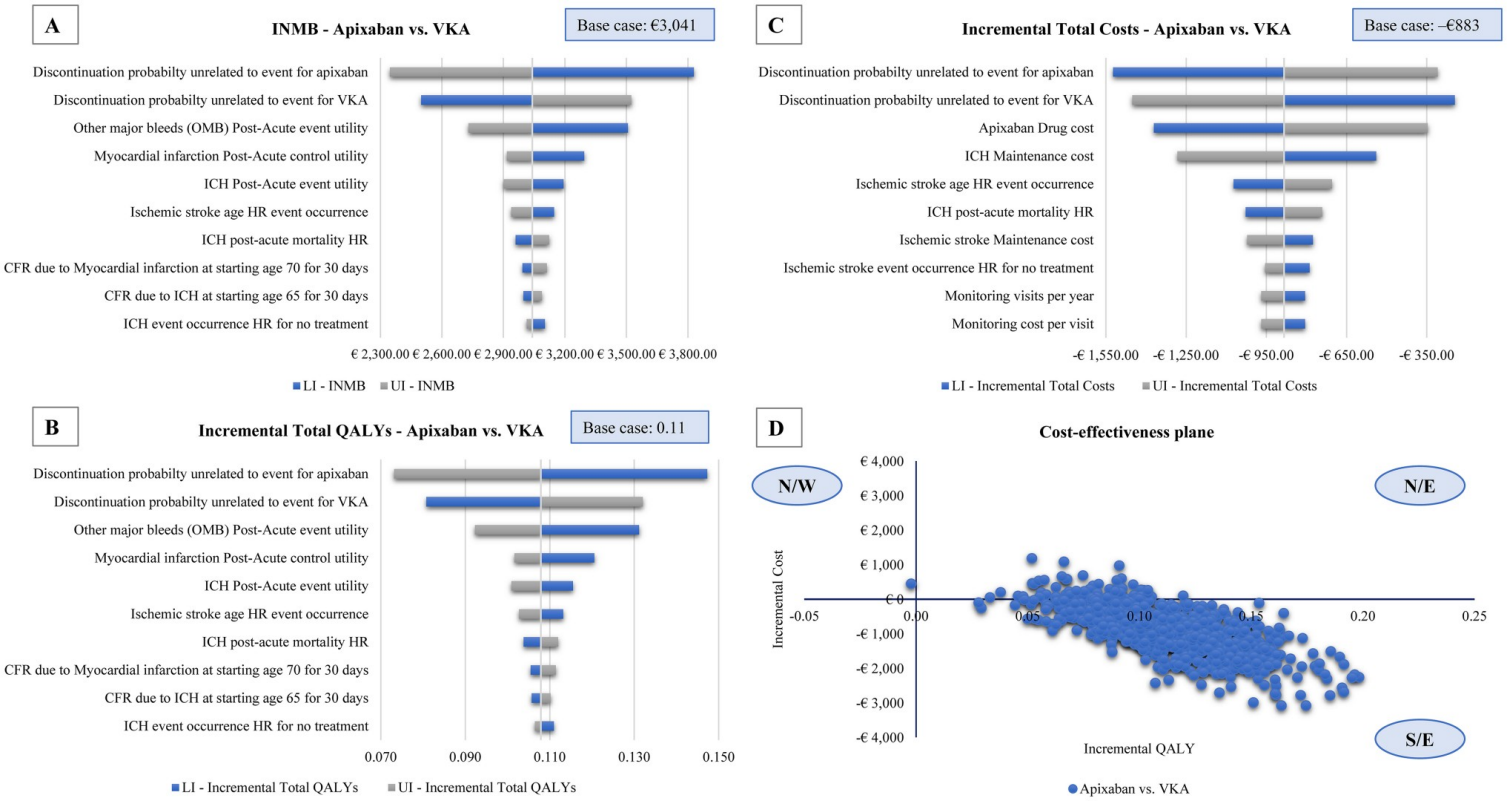
Results of extensive sensitivity analysis: INMB-DSA (A), incremental total QALYs-DSA (B), incremental total costs-DSA (C), and incremental costs and incremental QALYs-PSA (on cost-effectiveness plane) (D). Abbreviations: CFR = case fatality rate; DSA = deterministic sensitivity analysis; HR = hazard ratio; ICH = intracranial hemorrhage; INMB = incremental net monetary benefit; IS = ischemic stroke; LI = lower input value; MI = myocardial infarction; N/E = north-east; N/W = north-west; OMB = other major bleeds; PSA = probabilistic sensitivity analysis; QALYs = quality-adjusted life years; REV = urgent revascularization; S/E = south-east; UI = upper input value; VKA = vitamin K antagonist.

Results of DSA with respect to INMB (based on a WTP of €20,000 per QALY), incremental total QALYs, and incremental total costs for 10 most influential parameters to the outcomes are presented in Fig 1A–1C, respectively. Treatment discontinuation unrelated to events (for apixaban and VKA) and OMB post-acute event utility were the major drivers of uncertainty in INMB and incremental QALYs. Apixaban drug and ICH maintenance costs were the most influential parameters when incremental costs were considered. Compared with VKA, apixaban treatment led to cost savings (INMB: €2,346–€3,829; incremental total costs: €245–€1,523, per patient) as well as higher QALYs (incremental total QALYs: 0.073–0.147) across all ranges of parameters tested.

### Probabilistic sensitivity analysis

The PSA results are presented on the cost-effectiveness plane, where incremental QALYs and incremental costs of apixaban versus VKA are shown (Fig 1D). The majority of the simulations (92.6%) clustered in the South-East quadrant, indicating that apixaban was dominant (provided additional QALYs at lower costs than VKA). All but one (0.1%) remaining simulations (7.3%) were in the North-East quadrant, with all of them being below the €20,000 WTP threshold, thus indicating apixaban’s cost-effectiveness. Compared with base case, there was minimal deviation in the mean incremental costs (–€889 vs–€883), LYs (0.13 vs 0.13), and QALYs (0.11 vs 0.11). Hence, impact of each parameter uncertainty to the overall incremental benefit of apixaban versus VKA was limited. The cost-effectiveness acceptability curves (CEAC) (S4 Fig) indicated that apixaban appeared to be an optimal treatment option (>50%) across all WTP thresholds explored.

### Scenario analysis

Multiple scenarios were evaluated. The treatment strategy with apixaban was dominant over VKA in scenarios with shorter time horizon (10 or 20 years) than base case as well as when different treatment strategies and different switching timepoints were evaluated. Similar results were observed for all other scenarios analyzed (S2 Table).

Using warfarin unit costs instead of acenocoumarol unit costs had a limited impact on the base case results, with the incremental total costs (payer’s perspective) between apixaban and VKA decreasing from -€883 to -€824. The same limited impact of using warfarin unit cost was observed in PSA, with the majority of the PSA simulations (90.7%) clustered in the South-East quadrant, indicating that apixaban was dominant (provided additional QALYs at lower costs than VKA). All but one (0.1%) remaining simulations (9.2%) were in the North-East quadrant, with all of them being below the €20,000 WTP threshold, thus indicating apixaban’s cost-effectiveness.

When comparing the results across the three treatment strategies (see S1 Table—triple or dual therapy then monotherapy after 6 months, triple then dual therapy after 3 months then monotherapy at 12 months, dual therapy then monotherapy at 12 months), apixaban was associated with cost savings and greater LYs and QALYs vs. VKA, regardless of the treatment strategy chosen, from both the payer and societal perspectives. From the payer perspective, the treatment strategy used in the base case analysis (triple or dual therapy then monotherapy after 6 months) with apixaban resulted in being dominant compared to all other strategies with apixaban or VKA, while the “dual therapy then monotherapy” strategy with VKA was dominated by most of the other strategies and less costly-less effective vs. the “triple then dual then monotherapy” strategy with VKA. Similar results were observed when focusing on the societal perspective, with the base case treatment strategy with apixaban being dominant vs. most of the other strategies with apixaban or VKA, and cost-effective at a WTP €20,000 vs. “dual therapy then monotherapy” strategy with apixaban (ICER of €9,078). The “dual therapy then monotherapy” strategy with VKA remained either dominated or less costly-less effective vs. all other strategies with apixaban or VKA, as observed in the payer perspective analysis. However, note that the objective of this study was to compare economic implications between treatments (apixaban versus VKA) within each treatment strategy. The choice of treatment strategy itself (e.g., selection of the starting therapy and timing of step-down) should be driven by patient-specific considerations regarding ischaemic and bleeding risks rather than by cost considerations.

## Discussion

In this study, apixaban provided better long-term economic outcomes (with €883 cost savings per patient) and health outcomes (with 0.13 and 0.11 additional LYs and QALYs, respectively) than VKA. This cost savings associated with apixaban increased to €3,623 per patient, from the societal perspective, when IS and ICH indirect costs were considered. Better health outcomes and higher cost savings associated with apixaban than VKA were mostly attributed to lower bleeding (–13.9) and ischemic events (–1.8) per 100 patients over the simulated time horizon. Apixaban was cost-effective compared with VKA in 99.9% of the simulations (92.6% dominant and 7.3% cost-effective) of PSA and was dominant across all DSA and scenarios evaluated.

This study is the first, to the best of our knowledge, to investigate the costs and health outcomes associated with apixaban and VKA in patients with AF having ACS/PCI specific to the Spanish healthcare system. Hence, we compared our findings with the results from two analyses that evaluated cost-effectiveness of apixaban in preventing stroke and SE in the Spanish population with AF [25–27]. The models used in those two studies were broadly similar to our model in terms of clinical events considered (e.g., stroke, MI, bleeding, SE), although there are significant differences between the models (e.g., no joint health states modelled, no increased risk of events due to the event history, no REV considered, IS separated into mild, moderate, and severe). Nevertheless, findings of our analyses (with the base case modified to match in terms of cohort age, time horizon, and discounting) are in agreement with the results from both studies, with higher total costs (25–56%) and lower LYs and QALYs (14–23%) than in those two studies, due to higher number of bleedings and ischemic events in our study [25, 27]. These estimates are reasonable given that the modelled cohort in our study experienced ACS/PCI within 14 days, thus would require triple or dual therapy resulting in an increased risk of bleeding. Moreover, given the proximity to the index event, patients are expected to be at increased risk of subsequent ischemic events.

Assessing the generalizability of the AUGUSTUS trial population, on which the analysis is based, to the real-world Spanish AF population with ACS/PCI is challenging, given the lack of recent Spanish real-world studies on this specific population. Comparing the AUGUSTUS trial population to the Spanish AF population (with or without ACS/PCI) [47, 48, 53], highlighted comparable age and proportion of patients with history of hypertension, diabetes and stroke. Key differences were observed regarding gender distribution (in the AUGUSTUS trial over 70% of patients were males), history of heart failure (higher in the AUGUSTUS trial) and CHA_2_DS_2_-VASc/HAS-BLED scores (marginally higher in the AUGUSTUS trial), with the latter differences driven by the AUGUSTUS trial focus on AF patients who had a recent ACS and/or undergoing PCI, thus at higher risk of subsequent ischemic events.

Interestingly, the time in therapeutic range for patients receiving VKA in the AUGUSTUS trial (median time in therapeutic range of 59%) [20] was comparable to a recent real-world study in AF patients in Spain (mean time in therapeutic range of 58.3%-63.7%) [47]. In summary, the AUGUSTUS trial population characteristics seem comparable to the real-world Spanish AF population in terms of age and history of hypertension, diabetes and stroke while differences were noted in terms of gender distribution, history of heart failure and CHA_2_DS_2_-VASc/HAS-BLED scores. Further real-world studies specific to the Spanish AF population with ACS/PCI are needed for more adequate comparison.

Our analyses has several limitations. First, initial treatment strategy and timing of step-down are likely to vary in clinical practice, as per guideline recommendations [1, 12]. However, apixaban was dominant across all scenarios which included multiple relevant treatment strategies and switching points. Second, the model assumed that patients can only step-down (e.g. triple therapy->dual therapy->monotherapy–see S2 Table) and would not switch back (e.g. from monotherapy to triple therapy). While it is unclear whether this would often happen in clinical practice, it was not considered in the current model structure since it would have required to either add a significant number of health states or to change the model structure (i.e. using a patient-level simulation). In fact, the Markovian modelling approach handles time from the beginning of the simulation rather that time spent in a health state [54], with the second requirement needed if patients were allowed to also step-up therapy (i.e. the model would need to track the time that patients spend on each treatment strategy to know when to step-down after stepping-up). While this remains a limitation of the current model, it is important to note the following considerations. Not allowing patients to re-start triple/dual therapy or monotherapy after discontinuation, as in our model, is likely a conservative assumption for apixaban, since patients re-starting treatment would have further accrued the incremental difference in effectiveness between apixaban and VKA. In addition, re-starting dual/triple therapy after experiencing a clinical event while on monotherapy would only be applicable to a subset of the clinical events models (MI, ischemic stroke) since it is unlikely that patients experiencing an hemorrhagic event would switch back-up to double/triple therapy.

Third, duration of the AUGUSTUS trial [20] was limited to 6 months and only focused on triple and dual treatment strategies. Therefore, the monotherapy event rates, where possible, were sourced from a post-hoc analysis of data from the ARISTOTLE trial in population with CAD [30], raising potential inconsistencies if patient population in both trials were not similar. However, comparison of age and gender split ratio between the two trials (i.e., population with CAD and overall population) highlighted very similar characteristics. Fourth, long-term extrapolation of the ARISTOTLE trial [32] was required to run the simulation. Therefore, the current analyses assumed that the safety and efficacy of apixaban and VKA remained constant over time. This was an inherent limitation of all previous cost-effectiveness studies reviewed [25–27] that use short-term clinical evidence to inform long-term treatment effect. Hence, investigation of the efficacy of triple or dual therapy, followed by monotherapy in patients with AF having ACS/PCI in the real-word setting is warranted. Fifth, we assumed therapeutic equivalence of warfarin (used as VKA in the AUGUSTUS [20] and ARISTOTLE trials [32]) and acenocoumarol, the most common VKA in Spain, used for VKA drug cost in the current analyses. This assumption is common across all Spanish cost-effectiveness studies [25–27] and supported by recent clinical evidence [49]. Furthermore, using warfarin unit costs instead of acenocoumarol unit costs had a limited impact on the base case results and PSA results, suggesting that the current analysis results can be extended to other VKAs, as long as clinical effectiveness and unit costs are comparable to acenocoumarol and warfarin ones. Sixth, stroke rates from the AUGUSTUS trial were used as proxy for IS in our study. This is unlikely to significantly impact the analyses, since these rates were only limited to the first few cycles (3–12 months) and ICH rates were low. Other limitations were related to variability of data and different sources used for parameter inputs. While this is common across all cost-effectiveness studies, extensive sensitivity and scenario analyses confirmed the base case analysis results and conclusions. Finally, the model assumed that after a patient experienced two clinical events (e.g., IS and ICH), only the same subsequent events (e.g., either IS or ICH) could be experienced, thus not capturing the possibility of experiencing, for instance, an MI after an IS and ICH. This assumption was made to limit the model complexity and was based on the modelled elderly cohort.

## Conclusions

Apixaban was a dominant treatment strategy than VKA for patients with AF having ACS/PCI from both the Spanish payer’s and societal perspectives. Apixaban therapy was associated with lower costs and improved health outcomes, including a lower occurrence of bleeding and ischemic events. These findings were further corroborated by extensive sensitivity and scenario analyses, with apixaban being the dominant OAC, regardless of treatment strategy (triple/dual or combined) considered.

## Supporting information

S1 File Supplemental methods. (DOCX)

S2 File Supplementary data. (DOCX)

S1 Fig Overall model diagram. Blue boxes highlight long-term events, whereas green boxes highlight short-term events. Abbreviations: CAD = coronary artery disease; CABG = coronary artery bypass grafting; CRNMB = clinically relevant non-major bleeding; ICH = intracranial hemorrhage; IS = ischemic stroke; MI = myocardial infarction; OMB = other major bleeds; PCI = percutaneous coronary intervention; REV = urgent revascularization; SE = systemic embolism. ^a^PCI and CABG are the two accepted approaches for REV in CAD [1]. These two approaches were captured together in REV, with costs and consequences derived as weighted average between PCI and CABG. ^b^Represented severity of mild or moderate and severe in aggregate.
(TIF)

S2 Fig Model structure for single events. Abbreviations: ACS = acute coronary syndrome; AF = atrial fibrillation; PCI = percutaneous coronary intervention.
(TIF)

S3 Fig Model structure for subsequent events—Ischemic stroke example. Abbreviations: ICH = intracerebral hemorrhage; IS = ischemic stroke; MI = myocardial infarction; OMB = other major bleeds.
(TIF)

S4 Fig Probabilistic sensitivity analysis for apixaban versus VKA (on CEAC). Abbreviations: CEAC = cost-effectiveness acceptability curve; PSA = probabilistic sensitivity analysis; VKA = vitamin K antagonist; WTP = willingness-to-pay.
(TIF)

S1 Table Treatment strategies available in the cost-effectiveness model. Abbreviations: Dual = dual therapy; Mono = monotherapy; Triple = triple therapy; OAC = oral anticoagulant. ^a^OAC (apixaban, warfarin) + P2Y12 + aspirin. ^b^OAC (apixaban, warfarin) + P2Y12. ^c^OAC (apixaban, warfarin).
(DOCX)

S2 Table Scenario analysis description and results. (DOCX)
