# Supplementary material for: Cost-effectiveness analysis of apixaban versus vitamin K antagonists for antithrombotic therapy in patients with atrial fibrillation after acute coronary syndrome or percutaneous coronary intervention in Spain
Source: PLoS One. 2021 Nov 12;16(11):e0259251. doi: 10.1371/journal.pone.0259251 (PMC8589164; doi:10.1371/journal.pone.0259251)
Supplement: S1 File — (DOCX) [file pone.0259251.s001.docx]

# Supplemental File S1: Supplemental Methods

# Model diagram and model structure

The overall model structure is visualized in S1 Fig 1.

S1 Fig 1. Overall model diagram. Blue boxes highlight long-term events, whereas green boxes highlight short-term events.

Abbreviations: CAD = coronary artery disease; CABG = coronary artery bypass grafting; CRNMB = clinically relevant non-major bleeding; ICH = intracranial hemorrhage; IS = ischemic stroke; MI = myocardial infarction; OMB = other major bleeds; PCI = percutaneous coronary intervention; REV = urgent revascularization; SE = systemic embolism.

^a^PCI and CABG are the two accepted approaches for REV in CAD [1]. These two approaches were captured together in REV, with costs and consequences derived as weighted average between PCI and CABG.

^b^Represented severity of mild or moderate and severe in aggregate.

For each of the health states with long-term effect (intracranial hemorrhage [ICH], other major bleeds [OMB], ischemic stroke [IS], myocardial infarction [MI]), at the occurrence of the clinical event (e.g., i*schemic stroke* in S1 Fig 2), the patients moved to the corresponding acute health state (e.g., a*cute IS* in S1 Fig 2) for one model cycle, which was associated with an event specific case fatality rate (CFR), acute care cost and utility loss. Based on the CFR, patients then either moved to the corresponding post-acute health state or died. The post-acute health state was associated with excess mortality due to the history of clinical events and long-term disutility and disease management costs.

S1 Fig 2. Model structure for single events.

Abbreviations: ACS = acute coronary syndrome; AF = atrial fibrillation; PCI = percutaneous coronary intervention.

Once patients entered one of the post-acute health states (e.g., p*ost-acute IS* in S1 Fig 3), they either remained there or experienced any other clinical event (ICH, OMB, IS, MI) at each model cycle, transitioning to the corresponding acute health state (e.g., if a ICH was experienced, then patients moved to the acute *IS + ICH* health state). Note that it was assumed that a patient would only experience one clinical event at each model cycle, therefore concomitant clinical events within a model cycle were not captured. Furthermore, it was assumed that patients who experienced two clinical events, would only experience recurrence of the same two events (e.g., patients in the p*ost-acute ICH + IS* health state can only further experience *ICH* or *IS*). This assumption was adopted to avoid state-explosion, considering that a low proportion of patients were likely to experience more than two events and it was consistent with other published economic analyses [2].

The increased risk of experiencing a subsequent clinical event was captured through multiplicative hazard ratios derived from the literature, as in recently published economic models [3]. Furthermore, the model was flexible to also consider the increased risk of experiencing ICH, OMB, IS, or MI due to aging. Patients experiencing a second clinical event incurred an acute cost for the second event (e.g., patients with past MI experiencing an IS incurred IS acute cost). The combined management cost (long-term) for joint health states, accrued at each model cycle, was derived as the maximum of management costs for constitute events, as in previous economic studies [3, 4]. Similarly, patients experiencing a second clinical event incurred the acute event disutility for the acute event duration (e.g., patients experiencing an MI, following an IS would experience MI-specific reduction in quality of life for a pre-specified duration). The utility values for joint health states were calculated using a multiplicative method, as described in the National Institute for Health and Care Excellence (NICE) Decision Support Unit technical guidance [5]. Specifically, the joint health state utility value was derived by multiplying the baseline utility by the ratio of the utility for patients experiencing the event (e.g., MI) and patients not experiencing the event (from the control arm of the identified published literature used to derive the utility for patients experiencing the event).

S1 Fig 3. Model structure for subsequent events - ischemic stroke example.

Abbreviations: ICH = intracerebral hemorrhage; IS = ischemic stroke; MI = myocardial infarction; OMB = other major bleeds.

**References**

1. Spadaccio C, Benedetto U. Coronary artery bypass grafting (CABG) vs. percutaneous coronary intervention (PCI) in the treatment of multivessel coronary disease: quo vadis? -a review of the evidences on coronary artery disease. Ann Cardiothorac Surg. 2018 Jul;7(4):506-15.

2. Cowie MR, Lamy A, Levy P, Mealing S, Millier A, Mernagh P, et al. Health economic evaluation of rivaroxaban in the treatment of patients with chronic coronary artery disease or peripheral artery disease. Cardiovas Res. 2020;116(11):1918-24.

3. Sterne JA, Bodalia PN, Bryden PA, Davies PA, Lopez-Lopez JA, Okoli GN, et al. Oral anticoagulants for primary prevention, treatment and secondary prevention of venous thromboembolic disease, and for prevention of stroke in atrial fibrillation: systematic review, network meta-analysis and cost-effectiveness analysis. Health Technol Assess. 2017 Mar;21(9):1-386.

4. Pandor A, Pollard D, Chico T, Henderson R, Stevenson M. Rivaroxaban for preventing atherothrombotic events in people with acute coronary syndrome and elevated cardiac biomarkers: an evidence review group perspective of a NICE single technology appraisal. Pharmacoeconomics. 2016 May;34(5):463-77.

5. Ara R, Wailoo A. NICE Decision Support Unit Technical Support Documents. NICE DSU Technical Support Document 12: The Use of Health State Utility Values in Decision Models. London: National Institute for Health and Care Excellence (NICE) Copyright © 2011 National Institute for Health and Clinical Excellence, unless otherwise stated. All rights reserved.; 2011.
