# Supplementary material for: Cost-effectiveness analysis of apixaban versus vitamin K antagonists for antithrombotic therapy in patients with atrial fibrillation after acute coronary syndrome or percutaneous coronary intervention in Spain
Source: PLoS One. 2021 Nov 12;16(11):e0259251. doi: 10.1371/journal.pone.0259251 (PMC8589164; doi:10.1371/journal.pone.0259251)
Supplement: S2 File — (DOCX) [file pone.0259251.s002.docx]

# S2 File. Supplementary Data

**S2 Fig 1. Probabilistic sensitivity analysis for apixaban versus VKA (on CEAC).**

Abbreviations: CEAC = cost-effectiveness acceptability curve; PSA = probabilistic sensitivity analysis; VKA = vitamin K antagonist; WTP = willingness-to-pay.
