# Supplementary figures and images for: Cost-effectiveness analysis of apixaban versus vitamin K antagonists for antithrombotic therapy in patients with atrial fibrillation after acute coronary syndrome or percutaneous coronary intervention in Spain
Source: PLoS One. 2021 Nov 12;16(11):e0259251. doi: 10.1371/journal.pone.0259251 (PMC8589164; doi:10.1371/journal.pone.0259251)

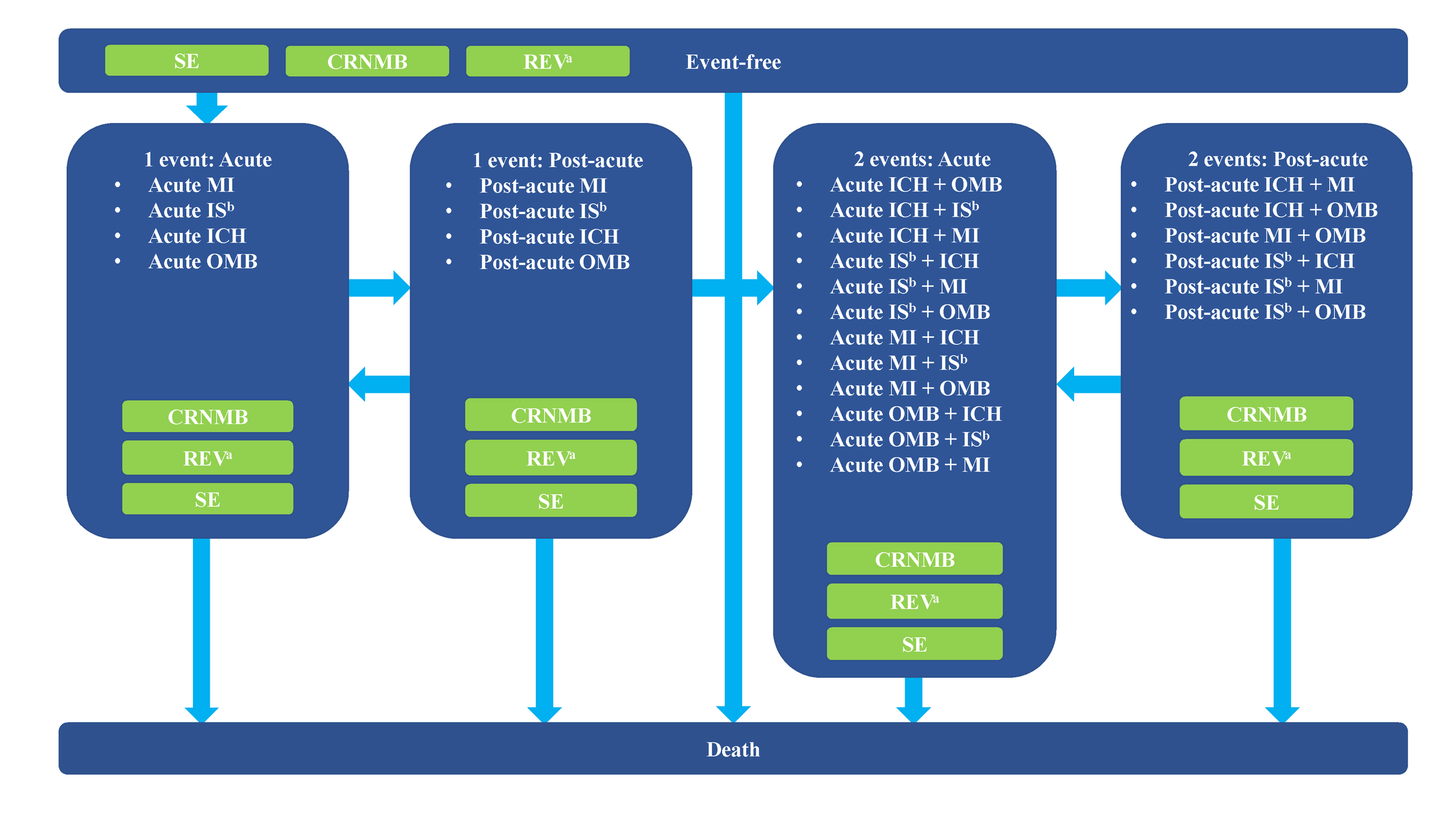

Supplement: S1 Fig — Abbreviations: CAD = coronary artery disease; CABG = coronary artery bypass grafting; CRNMB = clinically relevant non-major bleeding; ICH = intracranial hemorrhage; IS = ischemic stroke; MI = myocardial infarction; OMB = other major bleeds; PCI = percutaneous coronary intervention; REV = urgent revascularization; SE = systemic embolism. aPCI and CABG are the two accepted approaches for REV in CAD [1]. These two approaches were captured together in REV, with costs and consequences derived as weighted average between PCI and CABG. bRepresented severity of mild or moderate and severe in aggregate. (TIF) [file pone.0259251.s003.tif]

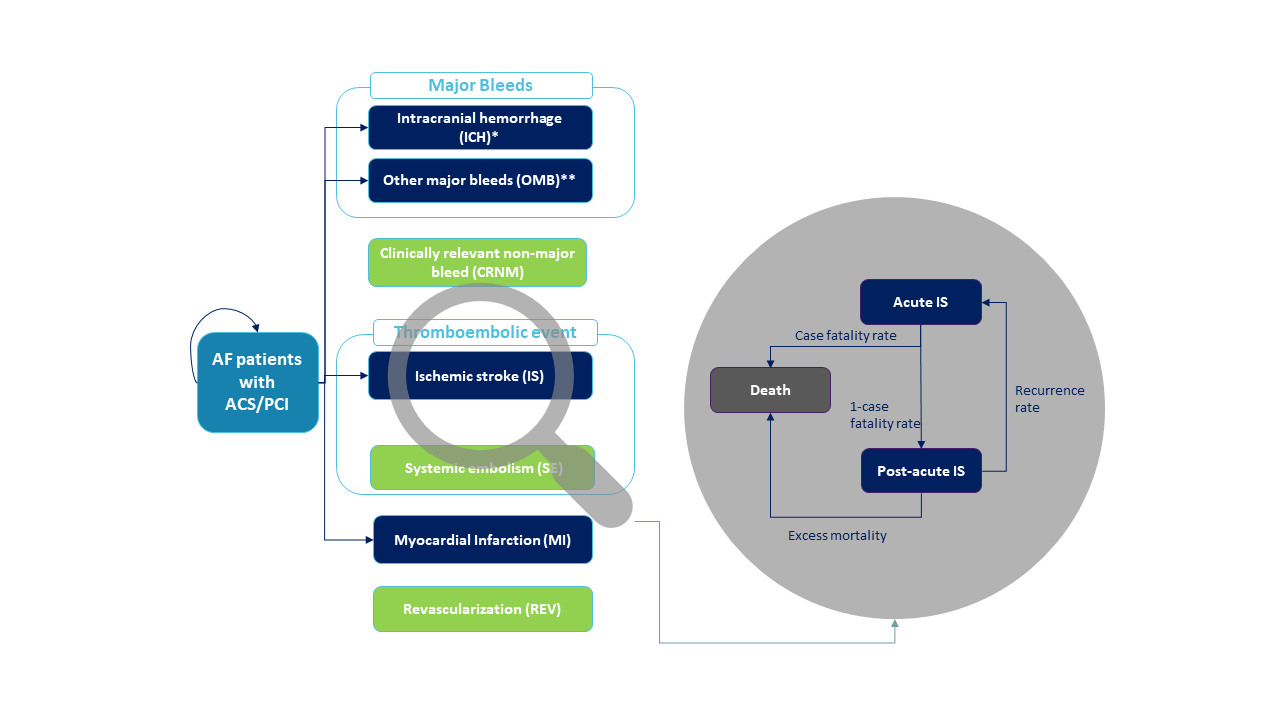

Supplement: S2 Fig — Abbreviations: ACS = acute coronary syndrome; AF = atrial fibrillation; PCI = percutaneous coronary intervention. (TIF) [file pone.0259251.s004.tif]

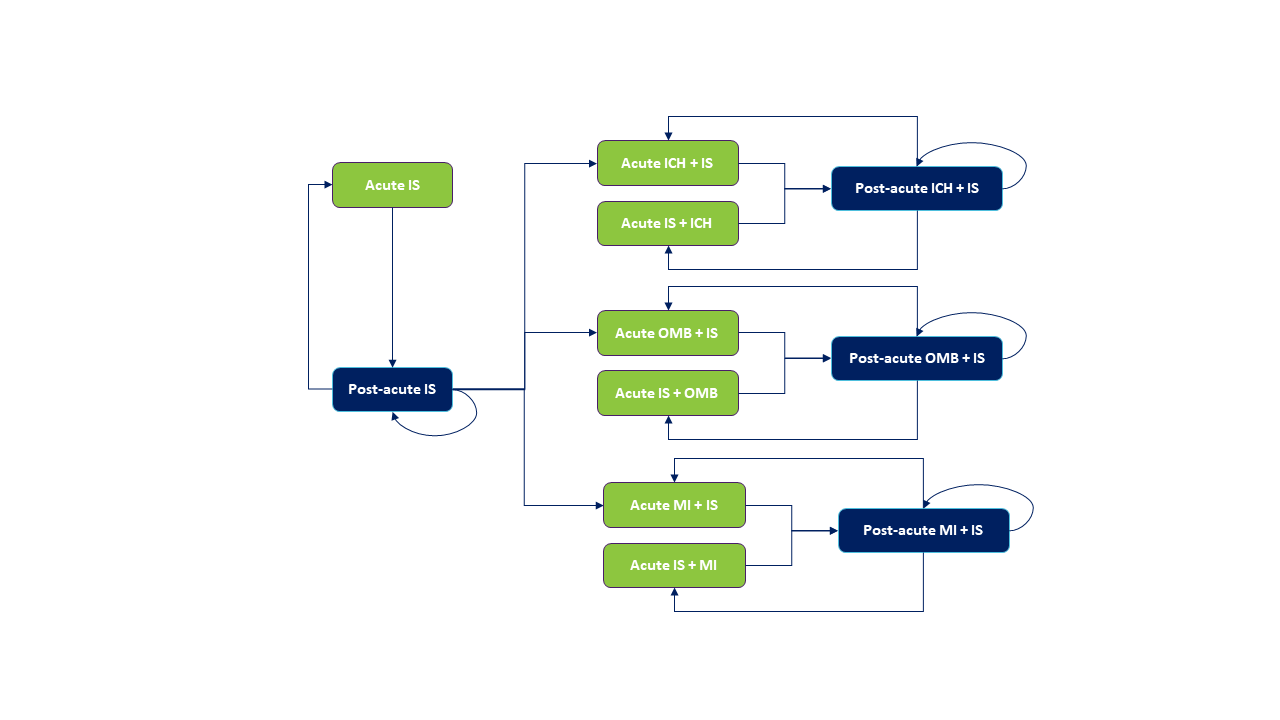

Supplement: S3 Fig — Abbreviations: ICH = intracerebral hemorrhage; IS = ischemic stroke; MI = myocardial infarction; OMB = other major bleeds. (TIF) [file pone.0259251.s005.tif]

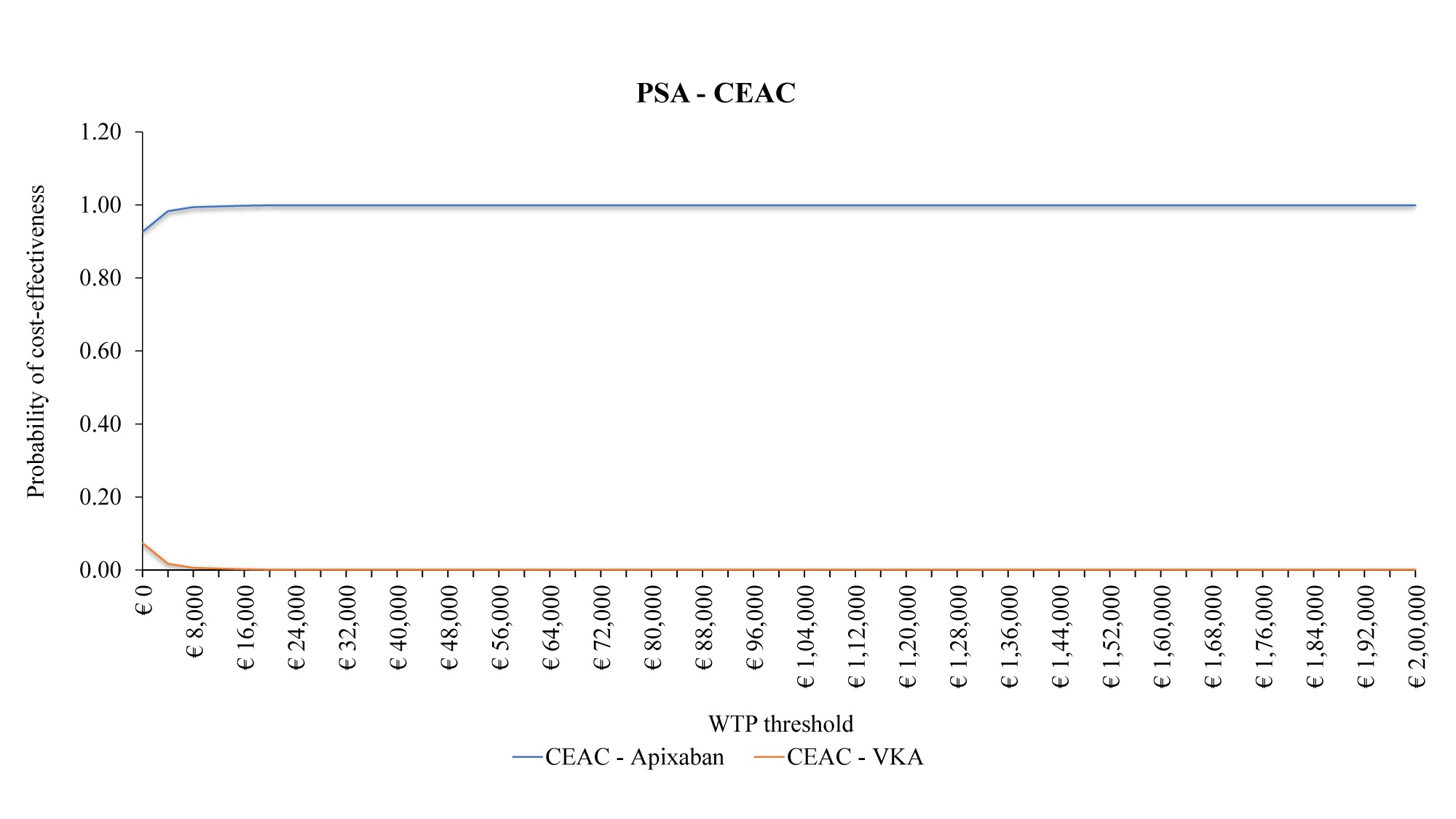

Supplement: S4 Fig — Abbreviations: CEAC = cost-effectiveness acceptability curve; PSA = probabilistic sensitivity analysis; VKA = vitamin K antagonist; WTP = willingness-to-pay. (TIF) [file pone.0259251.s006.tif]
