# Supplementary material for: Cost-effectiveness analysis of apixaban versus vitamin K antagonists for antithrombotic therapy in patients with atrial fibrillation after acute coronary syndrome or percutaneous coronary intervention in Spain
Source: PLoS One. 2021 Nov 12;16(11):e0259251. doi: 10.1371/journal.pone.0259251 (PMC8589164; doi:10.1371/journal.pone.0259251)
Supplement: S1 Table — Abbreviations: Dual = dual therapy; Mono = monotherapy; Triple = triple therapy; OAC = oral anticoagulant. aOAC (apixaban, warfarin) + P2Y12 + aspirin. bOAC (apixaban, warfarin) + P2Y12. cOAC (apixaban, warfarin). (DOCX) [file pone.0259251.s007.docx]

**S2 Table 1** **Treatment strategies available in the cost-effectiveness model.**

| Name | Definition |
| --- | --- |
| Triple^a^ or Dual^b^ 🡪 Mono^c^ | Patients start on triple or dual therapy and then switch to OAC monotherapy as per the AUGUSTUS trial. [1, 2] |
| Triple^a^ 🡪 Dual^b^ 🡪 Mono^c^ | Patients start on triple therapy and then switch to dual therapy and then to monotherapy, consistent with treatment guidelines. [3-5] |
| Dual^b^ 🡪 Mono^c^ | Patients start on dual therapy and then switch to monotherapy to investigate the AUGUSTUS trial arms, with and without aspirin, separately. |
|  |  |

Abbreviations: Dual = dual therapy; Mono = monotherapy; Triple = triple therapy; OAC = oral anticoagulant.

^a^OAC (apixaban, warfarin) + P2Y12 + aspirin.

^b^OAC (apixaban, warfarin) + P2Y12.

^c^OAC (apixaban, warfarin).

# References

1. Lopes RD, Heizer G, Aronson R, Vora AN, Massaro T, Mehran R, et al. Antithrombotic therapy after acute coronary syndrome or PCI in atrial fibrillation. N Engl J Med. 2019;380(16):1509-24. Epub 2019/03/19. doi: 10.1056/NEJMoa1817083. PubMed PMID: 30883055.

2. Lopes RD, Vora AN, Liaw D, Granger CB, Darius H, Goodman SG, et al. An open-Label, 2 × 2 factorial, randomized controlled trial to evaluate the safety of apixaban vs. vitamin K antagonist and aspirin vs. placebo in patients with atrial fibrillation and acute coronary syndrome and/or percutaneous coronary intervention: rationale and design of the AUGUSTUS trial. Am Heart J. 2018;200:17-23. Epub 2018/06/15. doi: 10.1016/j.ahj.2018.03.001. PubMed PMID: 29898844.

3. Capodanno D, Huber K, Mehran R, Lip GYH, Faxon DP, Granger CB, et al. Management of antithrombotic therapy in atrial fibrillation patients undergoing PCI: JACC state-of-the-art review. J Am Coll Cardiol. 2019;74(1):83-99. doi: 10.1016/j.jacc.2019.05.016.

4. Collet J-P, Thiele H, Barbato E, Barthélémy O, Bauersachs J, Bhatt DL, et al. 2020 ESC guidelines for the management of acute coronary syndromes in patients presenting without persistent ST-segment elevation: the task force for the management of acute coronary syndromes in patients presenting without persistent ST-segment elevation of the European Society of Cardiology (ESC). Eur Heart J. 2020. doi: 10.1093/eurheartj/ehaa575.

5. Hindricks G, Potpara T, Dagres N, Arbelo E, Bax JJ, Blomström-Lundqvist C, et al. 2020 ESC guidelines for the diagnosis and management of atrial fibrillation developed in collaboration with the European Association of Cardio-Thoracic Surgery (EACTS). Eur Heart J. 2020:ehaa612. Epub 2020/08/30. doi: 10.1093/eurheartj/ehaa612. PubMed PMID: 32860505.
