# Supplementary material for: Cost-effectiveness analysis of apixaban versus vitamin K antagonists for antithrombotic therapy in patients with atrial fibrillation after acute coronary syndrome or percutaneous coronary intervention in Spain
Source: PLoS One. 2021 Nov 12;16(11):e0259251. doi: 10.1371/journal.pone.0259251 (PMC8589164; doi:10.1371/journal.pone.0259251)
Supplement: S2 Table — (DOCX) [file pone.0259251.s008.docx]

**S2 Table 2 Scenario analysis description and results.**

| **Description** | **ICER per LY** | **ICER per QALY** | **Incremental total costs** | **Incremental LYs** | **Incremental QALYs** |
| --- | --- | --- | --- | --- | --- |
| **Payer perspective** |  |  |  |  |  |
| Default setup | Dominates | Dominates | –€883.42 | 0.13 | 0.11 |
| Use of a short time horizon (should capture lessened effects) | Dominates | Dominates | –€783.43 | 0.06 | 0.06 |
| Use of a longer (relative to previous scenario) time horizon (should capture increased effects, relative to previous scenario) | Dominates | Dominates | –€930.61 | 0.12 | 0.10 |
| Alternative treatment strategy where triple therapy is given separately from dual (3 months of triple, then 6 months of dual as per recent ESC guidelines [1, 2]) | Dominates | Dominates | –€1,156.10 | 0.14 | 0.12 |
| Alternative treatment strategy where triple therapy is given separately from dual (3 months of triple, then 9 months of dual as per recent ESC guidelines [1, 2]) | Dominates | Dominates | –€1,431.48 | 0.15 | 0.13 |
| Patients start on dual therapy and switch to monotherapy after 6 months, as per most recent ESC guidelines [1, 2] (switch to monotherapy in 6–12 months) | Dominates | Dominates | –€1,100.76 | 0.14 | 0.12 |
| Patients start on dual therapy and switch to monotherapy after 12 months, as per most recent ESC guidelines [1, 2] (switch to monotherapy in 6–12 months) | Dominates | Dominates | –€1,678.20 | 0.17 | 0.14 |
| All patients start without MI (event-free) | Dominates | Dominates | –€1,803.38 | 0.16 | 0.15 |
| All patients start with prior MI (no event-free patients in the cohort) | Dominates | Dominates | –€300.17 | 0.11 | 0.08 |
| The increase in event occurrence with age is not considered | Dominates | Dominates | –€820.91 | 0.12 | 0.10 |
| Discontinuation can only happen on occurrence of an event | Dominates | Dominates | –€749.43 | 0.30 | 0.24 |
| Higher discontinuation rates (100% for ICH, 30% for OMB, 30% for IS) [3] | Dominates | Dominates | –€946.03 | 0.13 | 0.11 |
| Alternative source using higher CFR for OMB (7.57%), likely includes deaths due to ICH [4] | Dominates | Dominates | –€783.67 | 0.14 | 0.12 |
| Alternative source using higher CFRs for MI (22–42%, three age groups), sourced from UK [5] | Dominates | Dominates | –€793.06 | 0.14 | 0.11 |
| Alternative source using higher CFR for IS (17.2%) [6] | Dominates | Dominates | –€ 802.28 | 0.13 | 0.11 |
| Patients off-treatment experiencing an event (ICH, OMB, IS, MI) accrue 2 weeks of treatment (triple or dual treatment) | Dominates | Dominates | –€856.75 | 0.13 | 0.11 |
| Same P2Y12 distribution between apixaban and VKA | Dominates | Dominates | –€877.99 | 0.13 | 0.11 |
| **Societal perspective** |  |  |  |  |  |
| Default setup for societal perspective | Dominates | Dominates | –€3,623.35 | 0.13 | 0.11 |
| Using Alvarez-Sabin et al. [7] estimates instead of Baron Esquivias 2014 [8] | Dominates | Dominates | –€3,928.01 | 0.13 | 0.11 |
| Using Alvarez-Sabin et al. [7] but only focusing on productivity losses estimates | Dominates | Dominates | –€950.10 | 0.13 | 0.11 |
| Including MI post-acute indirect costs, sourced from Delgado et al. [9] | Dominates | Dominates | –€2,478.85 | 0.13 | 0.11 |

Abbreviations: CFR = case fatality rate; Dual = dual therapy; ESC = European Society of Cardiology; ICER = incremental cost-effectiveness ratio; ICH = intracranial hemorrhage; IS = ischemic stroke; LYs = life year; MI = myocardial infarction; Mono = monotherapy; OMB = other major bleeds; PCI = percutaneous coronary intervention; QALY = quality-adjusted life year; Triple = triple therapy; UK = United Kingdom; VKA = vitamin K antagonist.

# References

1. Collet J-P, Thiele H, Barbato E, Barthélémy O, Bauersachs J, Bhatt DL, et al. 2020 ESC guidelines for the management of acute coronary syndromes in patients presenting without persistent ST-segment elevation: the task force for the management of acute coronary syndromes in patients presenting without persistent ST-segment elevation of the European Society of Cardiology (ESC). Eur Heart J. 2020. doi: 10.1093/eurheartj/ehaa575.

2. Hindricks G, Potpara T, Dagres N, Arbelo E, Bax JJ, Blomström-Lundqvist C, et al. 2020 ESC guidelines for the diagnosis and management of atrial fibrillation developed in collaboration with the European Association of Cardio-Thoracic Surgery (EACTS). Eur Heart J. 2020:ehaa612. Epub 2020/08/30. doi: 10.1093/eurheartj/ehaa612. PubMed PMID: 32860505.

3. Sterne JA, Bodalia PN, Bryden PA, Davies PA, Lopez-Lopez JA, Okoli GN, et al. Oral anticoagulants for primary prevention, treatment and secondary prevention of venous thromboembolic disease, and for prevention of stroke in atrial fibrillation: systematic review, network meta-analysis and cost-effectiveness analysis. Health Technol Assess. 2017;21(9):1-386. Epub 2017/03/11. doi: 10.3310/hta21090. PubMed PMID: 28279251; PubMed Central PMCID: PMCPMC5366855.

4. Chai-Adisaksopha C, Hillis C, Isayama T, Lim W, Iorio A, Crowther M. Mortality outcomes in patients receiving direct oral anticoagulants: a systematic review and meta-analysis of randomized controlled trials. J Thromb Haemost. 2015;13(11):2012-20. Epub 2015/09/12. doi: 10.1111/jth.13139. PubMed PMID: 26356595.

5. Vazquez-Oliva G, Zamora A, Ramos R, Marti R, Subirana I, Grau M, et al. Acute myocardial infarction population incidence and mortality rates, and 28-day case-fatality in older adults. The REGICOR study. Rev Esp Cardiol (Engl Ed). 2018;71(9):718-25. Epub 2017/11/28. doi: 10.1016/j.rec.2017.10.019. PubMed PMID: 29174866.

6. Marrugat J, Arboix A, García-Eroles L, Salas T, Vila J, Castell C, et al. [The estimated incidence and case fatality rate of ischemic and hemorrhagic cerebrovascular disease in 2002 in Catalonia]. Rev Esp Cardiol. 2007;60(6):573-80. Epub 2007/06/21. doi: 10.1157/13107113. PubMed PMID: 17580045.

7. Alvarez-Sabin J, Quintana M, Masjuan J, Oliva-Moreno J, Mar J, Gonzalez-Rojas N, et al. Economic impact of patients admitted to stroke units in Spain. Eur J Health Econ. 2017;18(4):449-58. Epub 2016/04/17. doi: 10.1007/s10198-016-0799-9. PubMed PMID: 27084749.

8. Baron Esquivias G, Escolar Albaladejo G, Zamorano JL, Betegon Nicolas L, Canal Fontcuberta C, de Salas-Cansado M, et al. Cost-effectiveness analysis comparing apixaban and acenocoumarol in the prevention of stroke in patients with nonvalvular atrial fibrillation in spain. Rev Esp Cardiol (Engl Ed). 2015;68(8):680-90. Epub 2014/12/17. doi: 10.1016/j.rec.2014.08.010. PubMed PMID: 25498373.

9. Delgado JF, Oliva J, Llano M, Pascual-Figal D, Grillo JJ, Comín-Colet J, et al. Health care and nonhealth care costs in the treatment of patients with symptomatic chronic heart failure in Spain. Rev Esp Cardiol (Engl Ed). 2014;67(8):643-50. Epub 2014/07/20. doi: 10.1016/j.rec.2013.12.014. PubMed PMID: 25037543.
